# Ocular structural remodeling and optic nerve damage in primary congenital glaucoma: findings from a vietnamese tertiary cohort

**DOI:** 10.3389/fmed.2026.1896417

**Published:** 2026-08-24

**Authors:** Thi Mai Anh Dao, Ngoc Son Nguyen, Van Khanh Tran, Thi Van Anh Bui

**Affiliations:** 1 Hanoi Medical University, Hanoi, Vietnam; 2 Department of Pediatric Ophthalmology, Vietnam National Eye Hospital, Hanoi, Vietnam; 3 Department of Ocular Imaging Diagnosis, Vietnam National Eye Hospital, Hanoi, Vietnam; 4 Center for Gene and Protein Research, Hanoi Medical University, Hanoi, Vietnam; 5 Department of Ophthalmology, Tam Anh General Hospital, Hanoi, Vietnam

**Keywords:** axial length, optic nerve damage, primary congenital glaucoma, retinal nerve fiber layer, ultrasound biomicroscopy

## Abstract

**Background:**

To characterize the clinical and ocular structural features of primary congenital glaucoma (PCG) and to identify factors associated with optic nerve damage in a Vietnamese tertiary referral cohort.

**Methods:**

In this cross-sectional study, 48 patients with PCG (85 affected eyes) examined at the Vietnam National Eye Hospital between 2022 and 2025 were included. Clinical, biometric, and ultrasound biomicroscopy (UBM) parameters were analyzed at both patient and eye levels. Descriptive comparisons were presented for all affected eyes and fellow healthy eyes, whereas inferential comparisons between affected and fellow healthy eyes were restricted to paired analyses of unilateral cases. Comparisons across C/D-based optic disk cupping categories were performed using patient-clustered generalized estimating equations (GEE) to account for inter-eye correlation. Multivariable GEE linear regression was used to identify factors independently associated with cup-to-disk ratio (C/D).

**Results:**

Among 48 patients (median age, 3.0 years), 37 (77.1%) had bilateral disease, leaving 11 patients with unilateral PCG and fellow healthy eyes available for paired comparisons; 37 of 46 evaluable patients (80.4%) were symptomatic at presentation. In paired analyses of these 11 unilateral cases, affected eyes demonstrated significantly larger corneal diameter (12.50 vs 10.50 mm, *p* = 0.001), longer axial length (25.40 vs 22.30 mm, *p* = 0.001), more myopic refractive error (−2.00 vs +0.38 D, *p* = 0.016), and greater C/D ratio (0.45 vs 0.00, *p* = 0.008) than fellow healthy eyes, whereas RNFL thickness did not differ significantly (*p* = 0.250); however, this comparison was based on only three paired OCT examinations and should be considered exploratory. UBM revealed wider anterior chamber angles, thinner limbal cornea, reduced iris thickness, longer ciliary processes, and deeper anterior chambers in affected eyes (all *p* < 0.05). Greater optic disk cupping was associated with more myopic refractive error (GEE *p* < 0.001), larger corneal diameter (GEE *p* < 0.001), higher intraocular pressure (GEE *p* = 0.002), and longer axial length (GEE *p* < 0.001). In multivariable GEE analysis, both corneal diameter (adjusted B = 0.106, 95% CI 0.025–0.187; *p* = 0.010) and axial length (adjusted B = 0.046, 95% CI 0.012–0.080; *p* = 0.008) remained independently associated with C/D ratio.

**Conclusion:**

Primary congenital glaucoma was associated with substantial structural alterations involving both the anterior and posterior segments of the eye. Among the evaluated structural parameters, axial length demonstrated the most consistent association with optic nerve damage across descriptive, severity-stratified, and multivariable analyses, whereas corneal diameter also remained independently associated after multivariable adjustment. These findings from this cross-sectional cohort suggest that axial elongation and corneal enlargement should be interpreted as structural markers associated with cumulative ocular remodeling and disease severity, rather than causal determinants of glaucomatous optic nerve damage. Longitudinal studies are needed to determine whether serial structural measurements, particularly axial length, can improve monitoring of disease progression in children with PCG.

## Introduction

Primary congenital glaucoma (PCG) is an uncommon but potentially blinding form of pediatric glaucoma caused by developmental abnormalities of the aqueous outflow system during early life. Although rare, with an estimated incidence ranging from 1 in 10,000 to 1 in 20,000 live births worldwide, PCG remains one of the most devastating causes of irreversible visual impairment in childhood because structural damage occurs during a critical period of ocular and visual development (1, 2). The burden of disease varies substantially across populations, with markedly higher incidence reported in regions where consanguinity is common, particularly in the Middle East, where rates may reach 1 in 2,500 live births (1). Clinically, PCG is bilateral in most patients, although unilateral disease has been reported in approximately 25%–30% of cases (1, 3). Importantly, visual disability in PCG extends beyond elevated intraocular pressure (IOP) alone, as prolonged ocular hypertension during infancy may disrupt normal globe growth, corneal transparency, axial elongation, and optic nerve development, leading to lifelong visual consequences even after IOP control is achieved.

The pathogenesis of PCG is primarily attributed to abnormal development of the anterior chamber angle, especially involving the trabecular meshwork and Schlemm’s canal, resulting in impaired aqueous humor outflow and sustained elevation of IOP (2, 4). Unlike adult glaucoma, in which structural rigidity of the mature eye limits deformation, the immature pediatric eye is highly susceptible to pressure-related biomechanical stretching under elevated IOP. Consequently, ocular enlargement and tissue stretching become characteristic features of PCG and may affect multiple anterior and posterior segment structures simultaneously. Throughout this manuscript, the term ocular structural remodeling is used as a descriptive umbrella term to denote the spectrum of secondary structural alterations observed in PCG, including globe enlargement (buphthalmos), corneal enlargement, axial elongation, anterior segment structural changes, and optic disk cupping. It is not intended to imply an intrinsic biological remodeling process independent of chronically elevated intraocular pressure but rather to describe the cumulative structural consequences of pressure-induced biomechanical stretching in the developing eye. Clinically, the disease commonly presents with epiphora, photophobia, and blepharospasm, accompanied by corneal enlargement, corneal edema, Haab’s striae, buphthalmos, axial elongation, and progressive optic nerve cupping (3, 5). Despite substantial advances in surgical management over recent decades, PCG continues to account for approximately 5%–18% of childhood blindness worldwide (6). Long-term visual outcomes are frequently compromised by amblyopia, corneal scarring, refractive abnormalities, and irreversible glaucomatous optic neuropathy (5, 7). Furthermore, optic nerve damage remains the principal determinant of permanent visual loss, yet the structural factors associated with glaucomatous damage in PCG remain incompletely characterized.

In recent years, increasing attention has been directed toward structural assessment in PCG, particularly the evaluation of anterior segment morphology and ocular biometric changes associated with disease severity. Ultrasound biomicroscopy (UBM) enables high-resolution visualization of structures that are difficult to evaluate using conventional clinical examination alone, including the anterior chamber angle, iris insertion, ciliary body, and zonular architecture (8). However, most previous studies have focused primarily on surgical outcomes or IOP control, whereas the relationship between anterior segment morphology, ocular biometry, and optic nerve damage has received comparatively limited attention. Moreover, available evidence has largely originated from small cohorts or highly selected populations, and comprehensive eye-level analyses integrating clinical, biometric, and UBM-derived parameters remain scarce.

These knowledge gaps are particularly important in low- and middle-income settings, where delayed diagnosis and restricted access to specialized pediatric glaucoma care may result in advanced disease at initial presentation. In Vietnam, published data on PCG remain limited, and there has been no comprehensive study integrating clinical findings with detailed anterior segment imaging and ocular biomarkers relevant to optic nerve injury. A better understanding of these secondary structural alterations and their relationship with optic nerve damage may improve risk stratification, facilitate earlier recognition of severe disease, and provide clinically meaningful insights into long-term visual prognosis in PCG. Therefore, this study aimed to characterize the clinical and structural features of primary congenital glaucoma and to identify factors associated with optic nerve damage in a Vietnamese tertiary referral cohort.

## Materials and methods

### Study design and participants

This was a hospital-based cross-sectional study conducted at a tertiary referral center, the Vietnam National Eye Hospital, between April 2022 and December 2025. The study population consisted of pediatric patients diagnosed with PCG who were examined during the study period. The diagnosis of childhood glaucoma was established in accordance with the consensus criteria of the Childhood Glaucoma Research Network (CGRN), which require the presence of at least two of the following features: elevated intraocular pressure (IOP), corneal enlargement or corneal edema with Haab’s striae, glaucomatous optic disk cupping, progressive myopia associated with abnormal ocular enlargement, or glaucomatous visual field defects. PCG was defined as childhood glaucoma occurring in the absence of identifiable secondary causes or associated ocular or systemic anomalies.

All patients with a confirmed diagnosis of PCG were considered eligible for inclusion, regardless of treatment status, including both newly diagnosed and previously treated cases. Patients were excluded if they had secondary glaucoma, concomitant ocular or systemic conditions that could affect ocular structure or function, or if informed consent could not be obtained from their legal guardians.

### Data collection

Data were systematically collected at both the patient level and the eye level. At the patient level, demographic and clinical variables included age at the time of examination, sex, laterality of disease (unilateral or bilateral), number of affected eyes, presenting subjective symptoms, and history of glaucoma surgery, defined as the presence of at least one previously operated eye.

At the eye level, detailed clinical and structural data were obtained. These included visual function assessed by best-corrected visual acuity (BCVA) and refractive status expressed as spherical equivalent. Anterior segment characteristics comprised corneal diameter, corneal clarity, and the presence of Haab’s striae. Intraocular pressure was recorded for each eye. Ocular biometry parameters included axial length. Posterior segment evaluation included the cup-to-disk ratio (C/D) and retinal nerve fiber layer (RNFL) thickness when measurable. In addition, anterior segment morphology was further characterized using ultrasound biomicroscopy (UBM), including quantitative measurements of anterior chamber angle at standardized clock-hour positions (3, 6, 9, and 12 o’clock), corneal thickness, iris thickness at 2 points IT1 and IT2 (IT1: measured at 1 mm from the pupil edge, IT2: measured at the mid-iris region), ciliary process length, zonular length, lens thickness, anterior chamber depth (ACD), and posterior chamber depth (PCD). Qualitative UBM features such as angle abnormalities, anterior iris insertion, and ciliary body insertion were also documented. For analytical purposes, eyes were categorized according to treatment status (untreated versus previously operated) and, in selected analyses, stratified by severity of optic nerve damage based on the cup-to-disk ratio. Affected eyes with gradable optic disks were categorized as having mild, moderate, or severe optic nerve damage according to cup-to-disk ratio thresholds used for severity stratification.

### Clinical assessment

All patients underwent a comprehensive ophthalmic examination according to standard clinical protocols. Intraocular pressure was measured using a rebound tonometer (Icare, Finland), which is commonly used for pediatric IOP assessment because it requires minimal cooperation and does not require topical anesthesia in many cases. Measurements were obtained during routine outpatient examination in cooperative children or during examination under general anesthesia when clinically indicated because adequate cooperation could not otherwise be achieved. Horizontal (transverse white-to-white) corneal diameter was measured using a Castroviejo caliper, during routine examination in cooperative children or during examination under general anesthesia when cooperation was insufficient, and axial length was determined by A-scan ultrasonography. Corneal status was classified into three predefined categories based on slit-lamp examination: clear cornea, corneal edema with partially obscured anterior segment structures, and opaque cornea with no visible anterior chamber details. The presence of Haab’s striae was assessed clinically during slit-lamp evaluation.

Visual function assessment was performed whenever feasible, taking into account patient age and cooperation. Refractive error was measured during routine clinical assessment and, whenever clinically feasible, cycloplegic refraction was performed. Refractive status was expressed as spherical equivalent (SE). The optic nerve head was evaluated by experienced ophthalmologists through dilated fundus examination whenever media clarity and patient cooperation permitted. Cup-to-disk ratio (C/D) was recorded when adequate visualization of the optic nerve head was possible. These parameters were used to characterize disease severity and structural damage associated with PCG.

### Ocular structural assessment

Retinal nerve fiber layer (RNFL) thickness was measured using optical coherence tomography (Cirrus HD-OCT 5000, Carl Zeiss Meditec, Dublin, CA, United States). Peripapillary RNFL measurements were obtained using the manufacturer’s standard optic disk scan protocol. Measurements were acquired only when image quality, media clarity, and patient cooperation allowed reliable acquisition. Scans were reviewed by experienced examiners, and images with motion artifacts, segmentation errors, poor centration, or inadequate signal quality that precluded reliable RNFL measurement were excluded from analysis.

Detailed anterior segment evaluation was performed using ultrasound biomicroscopy (VuMAX II, Sonomed Escalon, Lake Success, NY, United States) according to standardized imaging protocols. UBM was performed using a 50-MHz probe, allowing high-resolution visualization of anterior segment structures that may be difficult to assess clinically, were obtained at particularly in eyes with corneal opacity or limited cooperation. Radial scans the 3-, 6-, 9-, and 12-o’clock meridians according to the manufacturer’s recommended protocol. Quantitative UBM measurements included anterior chamber angle width at standardized clock-hour positions (3, 6, 9, and 12 o’clock), corneal thickness, iris thickness at two predefined locations (IT1, measured 1 mm from the pupillary margin under non-mydriatic conditions; and IT2, measured at the mid-iris region), ciliary process length, zonular length, lens thickness, anterior chamber depth (ACD), and posterior chamber depth (PCD), defined as the distance between the posterior iris surface and the anterior lens capsule. Although the 50-MHz probe provides excellent spatial resolution, its penetration depth is lower than that of lower-frequency probes. Consequently, visualization of very deep anterior segment structures in markedly enlarged PCG eyes may have been limited in some cases; however, only examinations judged technically adequate for the predefined measurements were included in the analysis.

In addition to quantitative metrics, qualitative structural features were systematically assessed, including angle abnormalities, anterior insertion of the iris, and abnormal insertion of the ciliary body. All OCT and UBM measurements were included only when judged to be technically reliable and interpretable. These parameters were used to characterize anterior segment morphology and to explore their relationship with optic nerve damage in primary congenital glaucoma.

### Data analysis

Statistical analyses were performed using SPSS version 20.0 (IBM Corp., Armonk, NY, United States). Continuous variables were summarized as mean ± standard deviation (SD) when normally distributed or as median with interquartile range (IQR) otherwise; normality was assessed using the Shapiro–Wilk test. Categorical variables were summarized as counts and percentages. For descriptive summaries of independent groups, continuous variables were presented as mean ± SD or median (IQR), and categorical variables as counts and percentages. For comparisons between all affected eyes and fellow healthy eyes, descriptive statistics only were presented because fellow healthy eyes were available exclusively in unilateral cases and therefore did not constitute a comparable independent control group. In unilateral primary congenital glaucoma, inferential comparisons between affected and fellow healthy eyes were restricted to within-patient paired analyses using the Wilcoxon signed-rank test for continuous variables and the exact McNemar test for paired binary variables. Because both eyes from the same patient could contribute to eye-level analyses, inferential analyses involving all affected eyes were performed using generalized estimating equations (GEE) to account for within-patient correlation. Patient identification was specified as the clustering variable, an exchangeable working correlation structure was assumed, and robust standard errors were used throughout. Gaussian GEE models with an identity link were applied for continuous outcomes and linear regression analyses, whereas binomial GEE models with a logit link were used for binary outcomes.

For analyses according to C/D-based optic disk cupping categories, affected eyes with gradable optic disks were classified as mild (C/D < 0.4), moderate (0.4 ≤ C/D < 0.7), or severe (C/D ≥ 0.7). These severity categories were defined according to cup-to-disk ratio thresholds commonly used for clinical severity stratification in pediatric glaucoma studies and expert consensus. Differences across these categories were evaluated using GEE models according to variable type.

Factors associated with cup-to-disk ratio were investigated using separate univariable GEE linear regression models, followed by a multivariable GEE linear regression model. Variables entered into the multivariable model were prespecified on the basis of clinical relevance, biological plausibility, and data completeness. Although refractive error was significantly associated with cup-to-disk ratio in the univariable analysis, measurements were available for only 25 eyes and were therefore not included in the primary multivariable model to avoid a substantial reduction in sample size and statistical precision. Accordingly, the primary model included intraocular pressure, corneal diameter, and axial length. History of glaucoma surgery was also excluded because it represents a post-diagnostic therapeutic intervention that may lie on the causal pathway between disease severity and the current ocular structural status, rather than a baseline determinant of optic nerve damage. Multicollinearity among predictors was assessed using variance inflation factors (VIFs) derived from the corresponding complete-case ordinary linear regression model, because conventional multicollinearity diagnostics are not directly available for GEE models. Regression coefficients (B) with 95% confidence intervals (CI) were reported. A prespecified one-eye-per-patient sensitivity analysis was additionally performed. For patients with unilateral PCG, the affected eye was included. For patients with bilateral PCG, one affected eye was randomly selected using a fixed random seed. The same multivariable linear regression specification was then applied to the one-eye-per-patient dataset to assess the robustness of the principal findings. Analyses were conducted using available-case data without imputation because several ophthalmic measurements were unavailable in a subset of eyes owing to limited cooperation, media opacity, or technical constraints. Data availability for the principal ophthalmic variables is summarized in Supplementary Table 1. A two-sided *p*-value < 0.05 was considered statistically significant. Given the exploratory nature of several subgroup and imaging analyses, no formal adjustment for multiple comparisons was applied. Therefore, these findings should be interpreted as hypothesis-generating rather than confirmatory.

## Results

A total of 48 patients with PCG were included, comprising 11 patients (22.9%) with unilateral disease and 37 patients (77.1%) with bilateral involvement. The median age at examination was 3.0 years (IQR, 0.0–5.75), with no significant difference between unilateral and bilateral cases (4.0 [IQR, 2.0–6.0] vs 3.0 [IQR, 0.0–5.5] years, *p* = 0.448). Sex distribution was balanced, with 24 males (50.0%) and 24 females (50.0%), and did not differ according to disease laterality (*p* = 1.000). Subjective symptoms at presentation were reported in 37 of 46 evaluable patients (80.4%) and tended to be more frequent among patients with bilateral disease than among those with unilateral disease (88.6% vs. 54.5%), although this difference did not reach statistical significance (*p* = 0.094). Surgical status was evaluated at the eye level. Among the 85 affected eyes, 41 eyes (48.2%) had undergone prior glaucoma surgery and 44 eyes (51.8%) had not undergone surgical intervention (Table 1).

**TABLE 1:** Patient-level demographic, disease pattern, treatment history, and genetic characteristics of the study population.

Characteristic	Overall (*N* = 48)	Primary congenital glaucoma		*P*-value
		Unilateral (*N* = 11)	Bilateral (*N* = 37)	
Age at examination, years	3.0 (0.0–5.75)	4.0 (2.0–6.0)	3.0 (0.0–5.5)	0.448
Sex				1.000
Male	24 (50.0)	6 (54.5)	18 (48.6)	–
Female	24 (50.0)	5 (45.5)	19 (51.4)	–
Disease laterality				0.025*
Unilateral PCG	11 (22.9)	11 (100.0)	0	–
Bilateral PCG	37 (77.1)	0	37 (100.0)	–
Subjective symptoms at presentation (*n* = 46)				0.094
Symptomatic	37 (80.4)	6 (54.5)	31 (88.6)	–
Asymptomatic / no subjective symptoms	9 (19.6)	5 (45.5)	4 (11.4)	–
History of glaucoma surgery (eye level)				–
No prior surgery	44 (51.8)			–
Prior surgery	41 (48.2)			–

Data are presented as median (IQR) or *n* (%). Subjective symptom data were available for 46 patients. History of glaucoma surgery was analyzed at the eye level (85 affected eyes), including 44 eyes without prior glaucoma surgery and 41 eyes with previous glaucoma surgery. *P*-values were calculated using the Mann–Whitney U test or Fisher’s exact test, as appropriate.

*p < 0.05 indicates statistical significance.

Among the 85 affected eyes, 44 (51.8%) had not undergone prior glaucoma surgery and 41 (48.2%) had undergone at least one surgical intervention. Best-corrected visual acuity was unmeasurable or unavailable in the majority of eyes (59/85, 69.4%), reflecting the challenges of visual function assessment in young children with PCG. Eyes with prior surgery were significantly more likely to have clear corneas than untreated eyes (65.9% vs. 22.7%), whereas corneal edema and opacity were more frequent among untreated eyes (overall *p* < 0.001). Haab’s striae were also observed more frequently in previously operated eyes than in untreated eyes (78.1% vs. 52.3%, *p* = 0.013). Previously operated eyes had significantly greater axial length than untreated eyes (*p* < 0.001). This finding most likely reflects that eyes requiring surgery represented a clinically more advanced subgroup with greater cumulative globe enlargement rather than an effect of surgical intervention itself. Intraocular pressure tended to be lower in previously operated eyes than in untreated eyes (19.76 ± 10.89 vs. 23.34 ± 11.02 mmHg), although the difference did not reach statistical significance (*p* = 0.078). Similarly, refractive error, cup-to-disk ratio, and RNFL thickness did not differ significantly between groups; however, interpretation of RNFL findings is limited by the very small number of available measurements, particularly in the untreated group (*n* = 1) (Table 2).

**TABLE 2:** Eye-level clinical and structural characteristics of affected eyes according to prior surgical status.

Characteristic	Overall (*n* = 85)	Prior surgical status		*P*-value
		No (*n* = 44)	Yes (*n* = 41)	
Visual function				<0.001*
Unmeasurable/not recorded	59 (69.4)	43 (97.7)	16 (39.0)	–
≥20/30	7 (8.2)	0	7 (17.1)	–
20/40–20/200	5 (5.9)	0	5 (12.2)	–
<20/200 to CF/low vision	14 (16.5)	1 (2.3)	13 (31.7)	–
Refractive error (SE), diopters (*n* = 25)	−1.50 (−2.25 to −0.50)	−3.75 (−5.13 to −1.31)	−1.38 (−2.00 to −0.50)	0.314
	(*n* = 25)	(*n* = 3)	(*n* = 22)	–
Corneal characteristics				
Corneal diameter (mm)	13.14 ± 1.12	13.01 ± 1.10	13.28 ± 1.13	0.271
Corneal status				<0.001*
Clear	37 (43.5)	10 (22.7)	27 (65.9)	–
Edematous	21 (24.7)	14 (31.8)	7 (17.1)	–
Opaque	27 (31.8)	20 (45.5)	7 (17.1)	–
Haab’s striae				0.013*
Present	55 (64.7)	23 (52.3)	32 (78.1)	–
Absent	30 (35.3)	21 (47.7)	9 (21.9)	–
Ocular enlargement and intraocular pressure				
Axial length (mm)	24.49 ± 2.54	23.40 ± 1.87	25.65 ± 2.66	<0.001*
Intraocular pressure (mmHg)	21.65 ± 11.04	23.34 ± 11.02	19.76 ± 10.89	0.078
Optic nerve/posterior segment				
Cup-to-disk ratio (C/D) (n = 58)	0.53 ± 0.35	0.53 ± 0.32 (*n* = 24)	0.58 ± 0.37 (*n* = 34)	0.470
RNFL thickness (μm) (n = 15)	70.07 ± 25.66	53^†^	71.29 ± 26.17 (*n* = 14)	–

Data are presented as mean ± SD or *n* (%).

† Only one eye was available; SD not applicable. Refractive error, cup-to-disk ratio, and RNFL thickness were available only in a subset of eyes. Comparisons were performed using the Student’s *t*-test, Mann–Whitney U test, or Fisher’s exact test, as appropriate.

*Significant at 0.05.

Table 3 summarizes the clinical and structural characteristics of 58 affected eyes with available cup-to-disk ratio (C/D) measurements, categorized into mild (C/D < 0.4), moderate (0.4 ≤ C/D < 0.7), and severe (C/D ≥ 0.7) C/D-based optic disk cupping groups. Ultrasound biomicroscopy (UBM) measurements were available for a subset of 33 eyes. Eyes with greater optic disk cupping demonstrated significantly more myopic refractive error, larger corneal diameter, longer axial length, and higher intraocular pressure (all GEE *p* ≤ 0.002). In contrast, corneal clarity, the presence of Haab’s striae, and prior glaucoma surgery did not differ significantly across the C/D-based groups. RNFL thickness also differed significantly among groups (GEE *p* < 0.001); however, this analysis was based on only 15 eyes with reliable OCT measurements and should therefore be considered exploratory. Among the UBM-derived quantitative parameters, only iris thickness at the IT1 point differed significantly across the three groups (GEE *p* = 0.014), with thinner values observed in eyes with more advanced cupping, whereas anterior chamber angle measurements, iris thickness at the IT2 point, zonular length, anterior chamber depth, posterior chamber depth, and all qualitative UBM features showed no significant associations with the degree of optic disk cupping (all GEE *p* > 0.05) (Table 3).

**TABLE 3:** Clinical and structural characteristics across C/D-based optic disk cupping categories, with patient-clustered comparisons.

Characteristic	Cup-to-disk (C/D) category			GEE *p*-value
	Mild (*n* = 17)	Moderate (*n* = 15)	Severe (*n* = 26)	
Clinical characteristics				
Prior glaucoma surgery, *n* (% of eyes within C/D category)	10 (58.8)	6 (40.0)	12 (46.2)	0.520
Refractive error, D	−1.25 (−2.00 to −0.38), *n* = 11	−0.25 (−1.13 to 2.13), *n* = 5	−3.75 (−5.63 to −1.38), *n* = 9	<0.001*
Intraocular pressure, mmHg	13.0 (10.0–22.5)	13.0 (11.0–17.0)	18.0 (13.0–33.0)	0.002*
Corneal diameter, mm	12.47 ± 0.86	12.97 ± 1.03	13.33 ± 1.22	<0.001*
Clear cornea, *n* (%)	13 (76.5)	6 (40.0)	18 (69.2)	0.167
Haab’s striae present, *n* (%)	14 (82.4)	7 (46.7)	19 (73.1)	0.267
Posterior segment characteristics				
Axial length, mm	23.98 ± 1.41	23.75 ± 1.73	25.76 ± 2.85	<0.001*
RNFL thickness, μm	84.5 (83.0–94.0), *n* = 4	65.0 (55.0–69.0), *n* = 3	69.0 (46.0–90.0), *n* = 8	<0.001*^†^
**Quantitative UBM parameters**	***n* = 12**	***n* = 6**	***n* = 15**	
ACA at 3 o’clock, degrees	41.3 (34.8–47.4)	38.8 (33.5–52.2)	40.9 (37.2–48.3)	0.981
ACA at 6 o’clock, degrees	42.5 (39.4–45.2)	39.9 (37.8–46.0)	38.0 (35.9–41.8)	0.137
ACA at 9 o’clock, degrees	43.5 (39.1–47.7)	38.5 (30.9–46.7)	40.2 (38.0–43.0)	0.969
ACA at 12 o’clock, degrees	41.2 (32.5–44.9)	43.9 (39.5–52.5)	42.8 (38.4–45.1)	0.224
Iris thickness, IT2, mm	0.277 ± 0.076	0.298 ± 0.037	0.255 ± 0.062	0.240
Iris thickness, IT1, mm	0.546 ± 0.075	0.580 ± 0.074	0.494 ± 0.112	0.014*
Zonular length, mm	1.33 (1.11–1.54)	1.73 (1.58–1.90)	1.42 (1.26–1.67)	0.078
Anterior chamber depth, mm	3.90 ± 0.35	3.97 ± 0.27	3.64 ± 0.51	0.946
Posterior chamber depth, mm	0.662 ± 0.105	0.727 ± 0.166	0.588 ± 0.175	0.062
Qualitative UBM parameters				
Angle abnormality, *n* (%)	7 (58.3)	2 (33.3)	7 (46.7)	0.607
Anterior iris insertion, *n* (%)	4 (33.3)	4 (66.7)	6 (40.0)	0.608
Abnormal ciliary body insertion, *n* (%)	1 (8.3)	2 (33.3)	3 (20.0)	0.587

Data are presented as mean ± standard deviation, median (interquartile range), or *n* (%), as appropriate. Inferential comparisons were performed using generalized estimating equations (GEE) with patient-level clustering, an exchangeable working correlation structure, and robust standard errors. Gaussian GEE models with an identity link were used for continuous variables, and binomial GEE models with a logit link for binary variables. Sample sizes varied because refractive error, RNFL, and UBM measurements were unavailable in some eyes. No adjustment for multiple comparisons was applied because these subgroup analyses were exploratory. ACA, anterior chamber angle; C/D, cup-to-disk ratio; GEE, generalized estimating equations; RNFL, retinal nerve fiber layer; UBM, ultrasound biomicroscopy.

*Statistically significant at *p* < 0.05.

† RNFL comparisons were based on only 15 eyes and should be interpreted as exploratory.

Table 4 presents both the descriptive comparison of all affected eyes and fellow healthy eyes and the paired within-patient comparison restricted to unilateral primary congenital glaucoma cases. Descriptively, affected eyes exhibited more myopic refractive error, larger corneal diameter, longer axial length, greater cup-to-disk ratio, and thinner retinal nerve fiber layer (RNFL) than fellow healthy eyes. Because fellow healthy eyes were available only in unilateral cases, inferential comparisons were restricted to paired analyses. Within the 11 unilateral patients, affected eyes had significantly more myopic refractive error, larger corneal diameter, longer axial length, and greater cup-to-disk ratio than their fellow healthy eyes (all *p* < 0.05), whereas intraocular pressure did not differ significantly (*p* = 0.287). RNFL thickness was lower in affected eyes but the difference was not statistically significant (*p* = 0.250); however, this comparison was based on only three paired OCT examinations and should therefore be interpreted cautiously. Among the paired UBM measurements, affected eyes demonstrated significantly wider anterior chamber angles at the 3 and 9 o’clock positions, thinner limbal cornea and iris thickness at the IT2 point, longer ciliary processes, and greater anterior chamber depth (all *p* < 0.05), whereas the remaining quantitative and qualitative UBM parameters showed no significant between-eye differences. These findings indicate substantial anterior segment remodeling associated with primary congenital glaucoma.

**TABLE 4:** Descriptive and paired within-patient comparisons between affected and fellow healthy eyes in unilateral primary congenital glaucoma.

Variable	All affected eyes (*n* = 85)	Fellow healthy eyes (*n* = 11)	*P*-value
A. Descriptive comparison			
Best-corrected visual acuity	7/26 (26.9%) 20/20–20/30 5/26 (19.2%) 20/40–20/200 14/26 (53.8%) < 20/200/qualitative	6/6 (100%) 20/20–20/30	–
Refractive error, D	−1.50 (−3.00 to −0.44) (*n* = 25)	0.38 (0.03 to 0.72) (*n* = 8)	–
Corneal diameter, mm	13.00 (12.00–14.00) (*n* = 85)	10.50 (10.00–11.50) (*n* = 11)	–
Axial length, mm	24.00 (22.65–25.85) (*n* = 85)	22.30 (21.70–23.00) (*n* = 11)	–
Intraocular pressure, mmHg	20.00 (13.00–27.50) (*n* = 85)	16.00 (12.00–17.00) (*n* = 11)	–
Cup-to-disk ratio	0.60 (0.30–0.90) (*n* = 58)	0.00 (0.00–0.30) (*n* = 11)	–
RNFL thickness, μm	83.00 (50.00–85.00) (*n* = 15)	100.00 (97.00–109.50) (*n* = 3)	–
**Variable**	**Affected eye**	**Fellow healthy eye**	***P*-value**
B. Paired within-patient comparison (11 unilateral patients)			
Refractive error, D	−2.00 (−3.75 to −1.19) (*n* = 7)	0.38 (0.09 to 0.66) (*n* = 8)	0.016*
Corneal diameter, mm	12.50 (12.00–12.75) (*n* = 11)	10.50 (10.25–11.25) (*n* = 11)	0.001*
Axial length, mm	25.40 (23.55–26.85) (*n* = 11)	22.30 (21.75–22.90) (*n* = 11)	0.001*
Intraocular pressure, mmHg	13.00 (13.00–31.50) (*n* = 11)	16.00 (12.50–16.50) (*n* = 11)	0.287
Cup-to-disk ratio	0.45 (0.15–0.88) (*n* = 10)	0.00 (0.00–0.25) (*n* = 11)	0.008*
RNFL thickness, μm	53.00 (30.50–74.00) (*n* = 3)	100.00 (97.00–109.50) (*n* = 3)	0.250
ACA at 3 o’clock, degrees	46.05 (42.35–64.45) (*n* = 8)	36.55 (34.65–45.60) (*n* = 8)	0.012
ACA at 6 o’clock, degrees	42.35 (36.75–45.00) (*n* = 8)	36.00 (34.25–46.15) (*n* = 8)	0.123
ACA at 9 o’clock, degrees	44.00 (42.15–48.65) (*n* = 8)	37.35 (32.25–42.80) (*n* = 8)	0.025
ACA at 12 o’clock, degrees	44.65 (43.20–46.35) (*n* = 8)	35.15 (30.90–39.95) (*n* = 8)	0.050
Central corneal thickness, mm	0.575 (0.535–0.615)	0.600 (0.565–0.620)	0.182
Limbal corneal thickness, mm	0.775 (0.670–0.900)	0.965 (0.870–1.035)	0.012
Iris thickness (IT2), mm	0.29 (0.23–0.34)	0.37 (0.27–0.43)	0.042
Iris thickness (IT1), mm	0.55 (0.42–0.62)	0.61 (0.57–0.72)	0.093
Ciliary process length, mm	0.92 (0.80–1.19)	0.79 (0.63–0.97)	0.042
Zonular length, mm	1.27 (1.13–1.38)	1.11 (1.03–1.29)	0.263
Lens thickness, mm	3.33 (3.13–3.67)	3.46 (3.40–3.62)	0.401
Anterior chamber depth, mm	3.81 (3.56–4.07)	2.98 (2.85–3.09)	0.012
Posterior chamber depth, mm	0.69 (0.62–0.72)	0.64 (0.49–0.66)	0.183
Angle abnormality	6/8 (75.0%)	3/8 (37.5%)	0.250
Anterior iris insertion	5/8 (62.5%)	1/8 (12.5%)	0.125
Ciliary body insertion to posterior iris surface	2/8 (25.0%)	4/8 (50.0%)	0.625

Part A presents descriptive summaries of all affected eyes and fellow healthy eyes; no inferential comparisons were performed because fellow healthy eyes were available only in unilateral cases. Part B presents paired within-patient comparisons in unilateral primary congenital glaucoma. Continuous variables are presented as median (interquartile range) and were compared using the Wilcoxon signed-rank test; paired categorical variables are presented as *n*/*N* (%) and were compared using the exact McNemar test. Sample sizes varied because OCT and UBM measurements were unavailable in some eyes. BCVA, best-corrected visual acuity; RNFL, retinal nerve fiber layer; UBM, ultrasound biomicroscopy.

*p < 0.05 indicates statistical significance.

**FIGURE 1:**
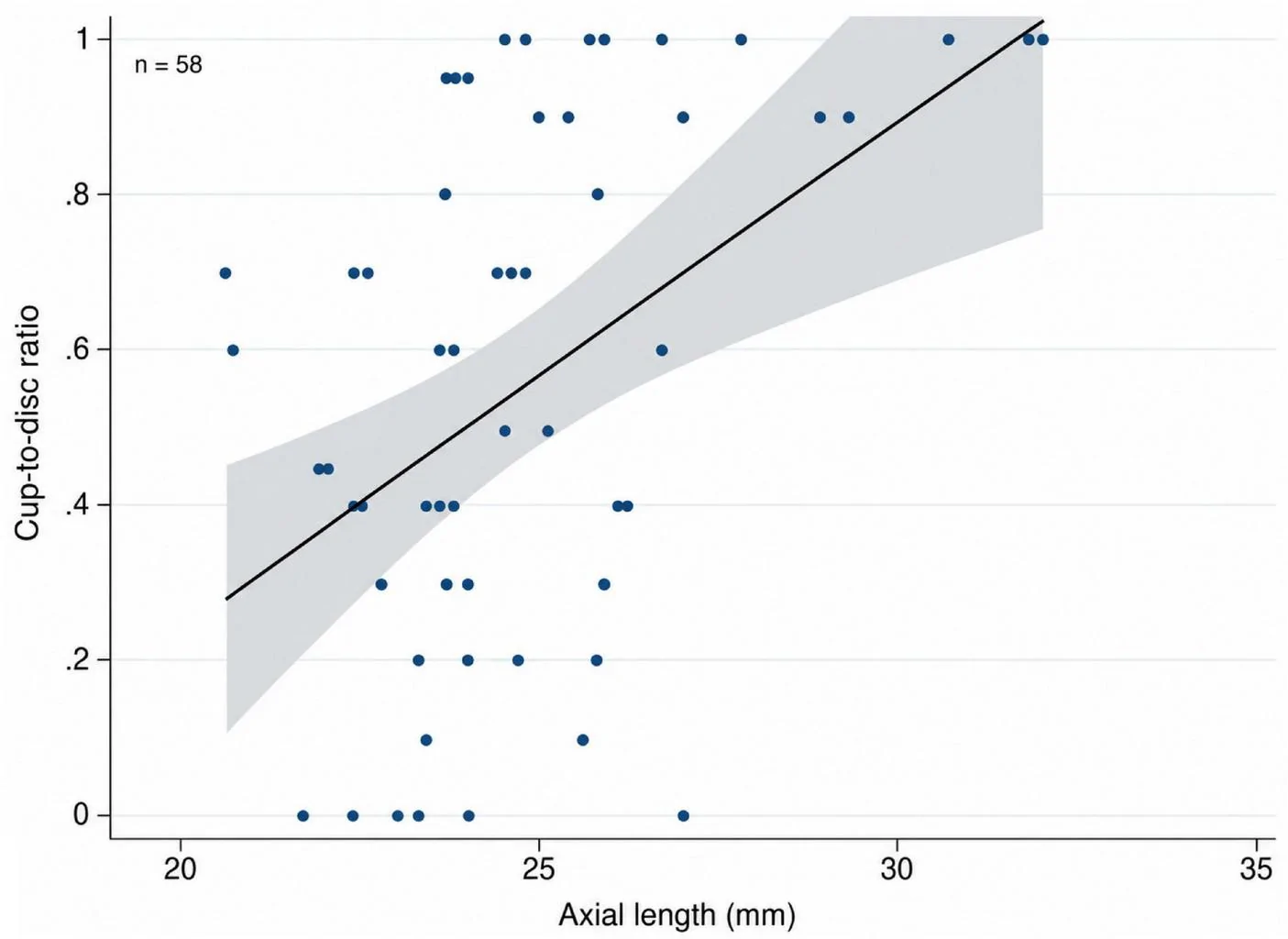
Association between axial length and cup-to-disc ratio in affected eyes with primary congenital glaucoma (*n* = 58). Each point represents one affected eye. The solid line indicates the fitted linear regression, and the shaded area represents the 95% confidence interval.

In the patient-clustered univariable GEE analyses, more myopic refractive error was associated with a greater cup-to-disk ratio (B = −0.075, 95% CI −0.129 to −0.021; *p* = 0.007). Larger corneal diameter was also associated with greater cupping (B = 0.147, 95% CI 0.069–0.225; *p* < 0.001), as was greater axial length (B = 0.071, 95% CI 0.035–0.108; *p* < 0.001). Intraocular pressure showed a positive but non-significant association with cup-to-disk ratio (B = 0.008, 95% CI −0.001 to 0.016; *p* = 0.086). None of the evaluated UBM-derived iris, zonular, or chamber-depth measurements was significantly associated with cup-to-disk ratio in the univariable models (Table 5). Figure 1 illustrates the positive association between axial length and cup-to-disk ratio, showing a progressive increase in optic disk cupping with greater axial length.

**TABLE 5:** Univariable GEE analysis of factors associated with cup-to-disk ratio in affected eyes.

Factor	Eyes, *n*	Coefficient (B)	95% CI	*P*-value
Refractive error, D	25	−0.075	−0.129 to −0.021	0.007*
Intraocular pressure, mmHg	58	0.008	−0.001 to 0.016	0.086
Corneal diameter, mm	58	0.147	0.069 to 0.225	<0.001*
Axial length, mm	58	0.071	0.035 to 0.108	<0.001*
Iris thickness, IT1, mm	33	−2.812	−7.992 to 2.367	0.287
Iris thickness, IT2, mm	33	−0.682	−2.057 to 0.694	0.331
Zonular length, mm	33	0.120	−0.099 to 0.339	0.284
Anterior chamber depth, mm	33	−0.047	−0.414 to 0.319	0.801
Posterior chamber depth, mm	33	−0.064	−0.598 to 0.471	0.816

Coefficients were estimated from separate univariable generalized estimating equation (GEE) linear models with cup-to-disk ratio as the dependent variable. Models accounted for within-patient correlation using an exchangeable working correlation structure and robust standard errors. Each model included all eyes with available data; therefore, the number of eyes varied across analyses. B represents the estimated mean change in cup-to-disk ratio per one-unit increase in the corresponding factor. CI, confidence interval; GEE, generalized estimating equations.

*Statistically significant at *p* < 0.05.

The multivariable GEE model included 58 affected eyes from 36 patients with complete data for cup-to-disk ratio, intraocular pressure, corneal diameter, and axial length. After adjustment for the other variables and within-patient correlation, both corneal diameter and axial length remained independently associated with cup-to-disk ratio. Each 1-mm increase in corneal diameter was associated with a 0.106-unit increase in cup-to-disk ratio (adjusted B = 0.106, 95% CI 0.025–0.187; *p* = 0.010), whereas each 1-mm increase in axial length was associated with a 0.046-unit increase in cup-to-disk ratio (adjusted B = 0.046, 95% CI 0.012–0.080; *p* = 0.008). Intraocular pressure measured at examination was not independently associated with cup-to-disk ratio after adjustment (adjusted B = 0.002, 95% CI −0.005 to 0.010; *p* = 0.556). Assessment of multicollinearity showed only low variance inflation factors for all included predictors (VIF range, 1.14–1.32), indicating no evidence of substantial multicollinearity (Table 6).

**TABLE 6:** Multivariable GEE analysis of factors associated with cup-to-disc ratio in affected eyes.

Factor	Adjusted B	95% CI	*P*-value	VIF^†^
Intraocular pressure, mmHg	0.002	−0.005 to 0.010	0.556	1.14
Corneal diameter, mm	0.106	0.025 to 0.187	0.010*	1.32
Axial length, mm	0.046	0.012 to 0.080	0.008*	1.30

Adjusted coefficients were estimated from a multivariable generalized estimating equation (GEE) linear model with cup-to-disk ratio as the dependent variable. The model included intraocular pressure, corneal diameter, and axial length and was based on 58 affected eyes from 36 patients, using an exchangeable working correlation structure (estimated within-patient correlation = 0.800). Adjusted B represents the estimated mean change in cup-to-disk ratio per one-unit increase in each predictor, adjusted for the remaining variables. VIFs were calculated from the corresponding complete-case ordinary linear regression model. CI, confidence interval; GEE, generalized estimating equations; VIF, variance inflation factor.

*Statistically significant at *p* < 0.05.

† Variance inflation factor.

## Discussion

In this hospital-based cross-sectional study, we characterized the clinical and structural ocular features of primary congenital glaucoma in a Vietnamese tertiary referral cohort and explored factors associated with optic nerve damage. Most patients presented with bilateral disease and substantial symptomatic burden, suggesting that primary congenital glaucoma in our setting is often recognized only after clinically significant disease has developed. Compared with fellow healthy eyes, affected eyes demonstrated consistent structural changes, including larger corneal diameter, longer axial length, more myopic refractive error, greater optic nerve cupping, thinner RNFL, and distinct anterior segment abnormalities on UBM. Importantly, while several clinical and structural parameters were associated with optic nerve damage in the univariable analyses, both corneal diameter and axial length remained independently associated with cup-to-disk ratio after multivariable GEE adjustment. Among these, axial length demonstrated the most consistent association across all analyses. This finding suggests that axial elongation may reflect the cumulative effects of disease duration, pressure-related biomechanical stress, age at onset, and ocular susceptibility, thereby serving as a structural marker of cumulative ocular remodeling rather than an independent determinant of optic nerve damage. Overall, these findings support the concept that primary congenital glaucoma is associated with widespread ocular structural remodeling that may already be advanced at the time of clinical presentation. To our knowledge, this study provides one of the first comprehensive clinical and imaging characterizations of ocular structural remodeling in Vietnamese children with primary congenital glaucoma by integrating clinical examination, ocular biometry, OCT, and UBM within a unified analytical framework. These findings complement existing evidence by evaluating multiple structural domains together and examining their independent associations with optic nerve damage.

The general clinical profile of our cohort is consistent with the established epidemiology of primary congenital glaucoma, in which bilateral involvement predominates and visual morbidity remains substantial despite advances in surgical care (7, 9, 10). In our series, 77.1% of patients had bilateral disease, closely matching the pattern reported in earlier reviews and large clinical series, in which bilateral involvement has been described in approximately 70%–80% of affected children (9, 10). However, beyond confirming known epidemiologic features, our data also carry an important contextual message: although the median age at examination was young, half of the patients had already undergone glaucoma surgery and symptomatic presentation was especially common in bilateral disease. In practice, this combination suggests that referral to tertiary care often occurs only after disease becomes clinically overt or structurally significant. This pattern is highly relevant in low- and middle-resource settings, where early recognition of subtle disease may be challenging and where tertiary hospitals preferentially receive more severe or complex cases. Thus, the present cohort may reflect the “real-world” clinical spectrum of primary congenital glaucoma that ophthalmologists in referral centers actually encounter, rather than an idealized early-screened population (7, 9).

One of the most informative findings of this study is the clear demonstration of global ocular enlargement in affected eyes. Compared with fellow healthy eyes, affected eyes had significantly larger corneal diameter, longer axial length, higher optic nerve damage and more myopic refractive error. These findings are pathophysiologically coherent with the classic understanding of infantile glaucoma, in which sustained elevation of IOP during early ocular development leads not only to optic nerve injury but also to enlargement and remodeling of the globe itself (2, 5). Corneal enlargement in early life is therefore reflect pressure-mediated tissue deformation during a vulnerable developmental window, while axial elongation and myopic shift may represent structural markers of cumulative disease burden (2). Al-Obaida et al. demonstrated that axial length in primary congenital glaucoma is closely related to age and IOP, reinforcing the concept that axial elongation captures the long-term impact of disease on the developing eye (11). Similar associations between increased axial length, globe stretching, and disease severity have also been emphasized in previous clinical studies of congenital glaucoma (12). In our cohort, this is particularly meaningful because axial length was greater not only in affected eyes versus fellow eyes, but also in previously operated eyes versus untreated eyes. Rather than implying a harmful effect of surgery, this finding more likely reflects that operated eyes represented a clinically more advanced subgroup with greater preexisting globe enlargement. Consistent with this interpretation, prior glaucoma surgery was not significantly associated with C/D severity in the patient-clustered analyses (Table 3). Therefore, surgical history was considered more likely to reflect previous disease severity and clinical management than an independent determinant of optic nerve damage in this cross-sectional cohort.

The corneal findings further support this interpretation. Corneal status differed significantly according to prior surgical status, with previously operated eyes being more likely to have clear corneas. This probably reflects partial reversal of corneal edema after IOP reduction, although structural consequences of prior globe distension may persist. deLuise and Anderson emphasized that in congenital glaucoma the immature cornea responds dynamically to elevated IOP, with enlargement, edema, and Haab’s striae reflecting the same underlying pathomechanism of tissue stretching (5). Previous clinicopathologic and imaging studies have likewise demonstrated that corneal changes in primary congenital glaucoma extend beyond simple enlargement and include structural disruption related to chronic biomechanical stress (13, 14). Our findings are consistent with this pathophysiological framework. In the paired within-patient comparison of unilateral cases, affected eyes demonstrated significantly larger corneal diameter than their fellow healthy eyes, supporting the concept that pressure-related globe enlargement is a characteristic structural manifestation of primary congenital glaucoma. In addition, Haab’s striae were observed exclusively in affected eyes, further reinforcing the close relationship between chronic ocular stretching and corneal remodeling. Similar observations have been consistently reported in large clinical series of primary congenital glaucoma, where corneal enlargement and Haab’s striae are among the most characteristic presenting signs of disease (7). Corneal diameter remained independently associated with cup-to-disk ratio after multivariable GEE adjustment, although the strength of this association was weaker than that observed for axial length. These findings suggest that corneal enlargement reflects cumulative biomechanical stretching and may provide complementary information regarding structural disease severity.

Another major strength of this study is the use of fellow healthy eyes in unilateral cases as an internal control. In pediatric glaucoma research, between-subject comparisons are often confounded by age, developmental stage, and measurement variability. By restricting inferential comparisons to paired within-patient analyses, our study minimizes confounding from patient-level characteristics and more convincingly isolates disease-related structural changes. Within these paired comparisons, affected eyes demonstrated significantly larger corneal diameter, longer axial length, greater optic nerve cupping, and more myopic refractive error than their fellow healthy eyes. Although RNFL thickness tended to be lower in affected eyes, the difference was not statistically significant in the paired analysis. However, this comparison was based on only three paired OCT examinations and should therefore be interpreted as exploratory. RNFL thinning in primary congenital glaucoma has been documented previously, including by Perucho-González et al., who reported significant RNFL reduction in children with primary congenital glaucoma compared with normal controls (15). Similar structural posterior segment abnormalities detected by OCT have also been described in pediatric glaucoma cohorts using both tabletop and handheld OCT systems (16, 17). However, interpretation of RNFL values in children requires caution, because normal pediatric RNFL is thicker than in adults and varies with age and imaging feasibility (18). Accordingly, although our paired RNFL analysis was limited by the small number of available OCT examinations, these findings should be considered exploratory. The observed trend was broadly consistent with previous reports and may suggest that structural posterior segment damage can be detected when high-quality OCT imaging is feasible. Therefore, our findings should be interpreted cautiously but are in keeping with previous evidence supporting the potential role of OCT in pediatric glaucoma assessment, while also highlighting the practical limitations of imaging in young children with poor cooperation or opaque media.

The UBM findings provide some of the most distinctive contributions of the present study. Compared with fellow healthy eyes, affected eyes showed wider anterior chamber angles at certain clock-hour positions, thinner limbal cornea, reduced iris thickness at the IT2 point, longer ciliary processes, and deeper anterior chamber depth. These results deserve careful interpretation. In primary congenital glaucoma, the problem is not a narrow angle in the adult primary angle-closure sense; rather, it is developmental dysgenesis of the angle structures, particularly the trabecular meshwork and Schlemm canal (19, 20). UBM therefore offers value not because it identifies simple angle crowding, but because it visualizes how anterior segment architecture is developmentally altered. High-frequency UBM imaging has been shown to be particularly useful for evaluating structures posterior to the iris and for assessing congenital angle abnormalities when corneal opacity or edema limits conventional optical visualization (21). Previous UBM studies in primary congenital glaucoma have consistently demonstrated abnormal iris insertion, deeper anterior chambers, altered angle configuration, and elongation or abnormal configuration of the ciliary body region (22). Our findings fit well within this literature and strengthen the argument that primary congenital glaucoma involves not only outflow pathway maldevelopment but broader anterior segment remodeling.

The apparent widening of the anterior chamber angle in affected eyes may seem counterintuitive if one assumes impaired outflow should correspond to a narrower angle. However, histopathologic and imaging studies have long shown that in congenital glaucoma the key abnormality is architectural dysgenesis of the trabecular meshwork and anterior chamber angle rather than geometric narrowing alone (23). As the globe enlarges, the anterior segment may become deeper and the angle may appear broader, while the outflow structures remain functionally abnormal. In this regard, our paired within-subject findings are particularly instructive: affected eyes had deeper anterior chambers and wider angles at 3 and 9 o’clock, yet they were clearly the diseased eyes with larger corneas, longer axial lengths, and more severe optic nerve cupping. Similar observations have been reported in UBM-based studies of primary congenital glaucoma, in which enlarged ocular dimensions coexisted with widened anterior chamber angles despite persistent outflow dysfunction (24). This dissociation between apparent angle width and effective outflow function is clinically important. It suggests that UBM-derived angle width should not be interpreted simplistically as evidence of preserved drainage; rather, it must be contextualized within the developmental pathology of primary congenital glaucoma.

The findings related to the iris, limbus, and ciliary body also merit deeper discussion. Affected eyes had thinner limbal cornea, thinner iris at the IT2 point, and longer ciliary processes than fellow healthy eyes. These changes likely reflect a combination of developmental dysgenesis and secondary mechanical stretching as the globe enlarges under elevated IOP. Experimental and anatomic studies have emphasized the structural and functional importance of the ciliary body and iris configuration in aqueous humor dynamics and anterior segment development (25). Alterations in ciliary process morphology may also influence aqueous humor dynamics, potentially contributing to disease progression. Previous UBM-based investigations in primary congenital glaucoma have similarly demonstrated abnormal iris insertion, altered ciliary process morphology, and remodeling of the anterior segment associated with globe enlargement (26, 27). In our cohort, the presence of these changes supports the concept of primary congenital glaucoma as a disease of whole anterior segment remodeling rather than isolated trabecular malfunction. Nevertheless, most qualitative UBM features were not significantly associated with optic nerve damage severity. This is an important negative finding. It suggests that while UBM is highly informative for disease phenotyping and structural characterization, the anatomic abnormalities it reveals may be more reflective of the developmental substrate of primary congenital glaucoma than of the degree of subsequent glaucomatous neuropathy.

The analysis according to three levels of optic nerve damage severity offers further insight into the disease course. Eyes with more advanced optic nerve damage had significantly more myopic refraction, and IOP showed a significant increasing trend with greater severity. Axial length also showed an increasing trend. These patterns are biologically plausible and clinically coherent. Myopic shift and axial elongation likely represent accumulated deformation over time, while higher IOP at examination may indicate ongoing insufficient control in the eyes with greatest structural damage. Similar associations between axial elongation, refractive change, and disease severity have been described in previous studies of primary congenital glaucoma and pediatric glaucoma progression (11, 28). However, GEE-based comparisons showed no significant differences in these parameters between eyes with mild and moderate optic disk damage and that many UBM parameters did not differ across severity groups underscores an important principle: optic nerve injury in primary congenital glaucoma is multifactorial and temporally cumulative. Observed optic disk cupping in primary congenital glaucoma likely reflects the cumulative effect of longstanding ocular stretching, chronic pressure-related burden, age at onset, disease duration, timing of surgery and tissue susceptibility, rather than isolated measurements obtained at a single examination. A single cross-sectional measurement may therefore incompletely capture the mechanisms that produced the observed disk cupping.

This interpretation is supported by the regression analyses. In the patient-clustered univariable GEE analyses, more myopic refractive error, longer axial length, and larger corneal diameter were associated with greater optic nerve damage. These findings suggest that both global globe enlargement and selected anterior segment geometry may track with disease severity. However, after multivariable GEE adjustment accounting for inter-eye correlation, both larger corneal diameter and longer axial length remained independently associated with greater cup-to-disk ratio, whereas intraocular pressure was no longer independently associated with optic nerve damage. Although both structural parameters remained significant, axial length demonstrated the most consistent association across the descriptive comparisons, C/D-based severity analyses, and multivariable regression models, supporting its potential role as an integrated structural marker of cumulative disease burden in PCG. Corneal diameter may similarly reflect cumulative biomechanical remodeling of the developing globe, although its association appeared less consistent across analyses. The low variance inflation factors observed in the multivariable model suggest that the independent associations of axial length and corneal diameter were unlikely to be explained by problematic multicollinearity, despite both variables representing related aspects of globe enlargement. Nevertheless, the consistent direction of association observed for axial length and intraocular pressure across multiple analyses supports the concept that cumulative ocular enlargement and chronic pressure-related stress contribute importantly to optic nerve remodeling in congenital glaucoma. In pediatric glaucoma, this interpretation is clinically plausible because IOP fluctuates, may be influenced by cooperation or anesthesia, and in previously operated eyes may underestimate prior disease burden (29). In addition, rebound tonometry measurements may be influenced by altered corneal biomechanics and corneal enlargement in primary congenital glaucoma, which could introduce measurement variability despite the widespread clinical use of this technique in pediatric practice. Similarly, axial elongation and myopic refractive shift may represent structural footprints of longstanding biomechanical stress, as previously described in studies evaluating ocular enlargement and refractive change in primary congenital glaucoma (12).

Our data do not diminish the role of IOP in primary congenital glaucoma pathophysiology; instead, they suggest that in cross-sectional datasets, IOP measured at a single visit is an imperfect surrogate for the cumulative injurious load borne by the optic nerve. Consistent with this interpretation, intraocular pressure was not independently associated with cup-to-disk ratio in the multivariable GEE model after adjustment for corneal diameter and axial length. This finding should not be interpreted as evidence against the pathogenic importance of IOP, but rather as reflecting the limitation of a single cross-sectional measurement in capturing the cumulative pressure exposure experienced by the developing eye. With a larger sample size, longitudinal follow-up, and repeated IOP measurements over time, the relationship between long-term IOP burden and optic nerve damage might become more apparent.

The present findings may have practical implications for the follow-up of children with primary congenital glaucoma. In many pediatric patients, reliable visual acuity testing, visual field assessment, and even detailed optic nerve evaluation may be limited by poor cooperation, young age, or corneal opacity. In contrast, axial length measurement is objective, reproducible, and widely available in routine clinical practice. Serial monitoring of axial growth may therefore provide complementary information regarding disease severity and structural progression, particularly in settings where advanced imaging modalities or functional testing are difficult to obtain. At the same time, the observed independent association between corneal diameter and cup-to-disk ratio suggests that corneal enlargement may provide complementary structural information when interpreted together with axial length rather than as an alternative marker of disease severity. Nevertheless, because the present study was cross-sectional, these structural parameters should be regarded as markers of cumulative ocular remodeling rather than independent determinants of glaucomatous damage. Although longitudinal studies are required to validate this approach, our findings suggest that axial length and, to a lesser extent, corneal diameter may serve as useful adjunctive structural biomarkers in the clinical assessment of childhood glaucoma. Rather than introducing entirely new structural markers, our findings provide an integrated evaluation of established ocular parameters and clarify their relative contributions to optic nerve damage within a single clinically well-characterized cohort.

This study has several limitations that should be acknowledged. First, the cross-sectional design precludes assessment of longitudinal structural progression and limits causal inference regarding factors associated with cup-to-disk ratio. Second, the sample size was relatively modest, particularly for subgroup analyses and variables with incomplete availability such as RNFL thickness and UBM measurements. In particular, the RNFL analyses were based on only a small subset of eyes and should therefore be considered exploratory. Third, although eye-level analyses included both eyes from some patients, within-patient correlation was addressed using generalized estimating equations with robust standard errors. Nevertheless, the relatively small number of patients and incomplete availability of several imaging variables may still have reduced statistical power and the precision of some subgroup estimates. Accordingly, statistically significant findings from subgroup and imaging analyses should be interpreted cautiously and considered exploratory pending confirmation in larger prospective cohorts. In addition, the multivariable analyses were based on complete-case data because several ophthalmic measurements were unavailable in a subset of eyes, which may have introduced some loss of precision. Fourth, several ophthalmic variables had incomplete data because of limited cooperation in young children, media opacity, and the practical challenges of obtaining high-quality OCT and UBM measurements. Finally, because this was a tertiary referral center-based cohort, the study population may have been enriched for more severe or complex cases, potentially limiting generalizability to milder community-based primary congenital glaucoma populations. In addition, intraocular pressure was measured at a single examination using rebound tonometry. Although this technique is widely used in pediatric ophthalmology because it requires minimal cooperation and generally does not require topical anesthesia, the measurements may nevertheless have been influenced by patient cooperation, examination under general anesthesia in some children, and altered corneal biomechanics associated with primary congenital glaucoma. Accordingly, a single IOP measurement should be interpreted cautiously, as it may not fully represent cumulative pressure exposure over the course of the disease. Finally, interpretation of the structural outcome also warrants caution because optic disk cupping in primary congenital glaucoma may partially reflect reversible posterior bowing of the lamina cribrosa in addition to true axonal loss. Accordingly, the present cross-sectional study used cup-to-disk ratio as a structural clinical marker rather than a direct measure of irreversible optic nerve injury.

## Conclusion

In this Vietnamese cohort of primary congenital glaucoma, both axial length and corneal diameter remained independently associated with optic nerve damage after multivariable GEE adjustment accounting for inter-eye correlation. Among the evaluated structural parameters, axial length demonstrated the most consistent association across the descriptive, C/D-based severity, and multivariable analyses, whereas intraocular pressure and most anterior segment parameters demonstrated weaker or non-significant relationships. These findings from this cross-sectional cohort suggest that axial elongation and corneal enlargement should be interpreted as structural markers associated with cumulative ocular remodeling and disease severity, rather than causal determinants of glaucomatous optic nerve damage. Clinically, these results support incorporating axial length and corneal diameter as complementary structural markers in the assessment of disease severity, together with anterior segment dysgenesis and intraocular pressure. Such an approach may be particularly valuable in pediatric patients, in whom functional assessment is often limited by young age, poor cooperation, or media opacity. However, longitudinal studies are required to determine whether serial structural measurements, particularly axial length, can be used to monitor disease progression or improve risk stratification in primary congenital glaucoma.

## Data Availability

The raw data supporting the conclusions of this article will be made available by the authors, without undue reservation.
