# Supplementary material for: Ocular structural remodeling and optic nerve damage in primary congenital glaucoma: findings from a vietnamese tertiary cohort
Source: Front Med (Lausanne). 2026 Aug 24;13:1896417. doi: 10.3389/fmed.2026.1896417 (PMC13547039; doi:10.3389/fmed.2026.1896417)
Supplement: Supplementary file 1 [file Table_1.DOCX]

Supplementary Table S1. Availability of ophthalmic measurements across affected eyes

| **Variable** | **Eyes with data** | **Missing** | **Main reason for unavailable measurements** |
| --- | --- | --- | --- |
| Visual acuity | 26 | 59 | Young age / poor cooperation |
| Refraction | 25 | 60 | Poor cooperation |
| Cup-to-disc ratio | 58 | 27 | Corneal opacity or inadequate fundus visualization |
| RNFL thickness | 15 | 70 | Poor cooperation, media opacity, or inadequate OCT image quality |
| UBM quantitative measurements | 33 | 52 | Limited cooperation or technically inadequate examination |

Missing data primarily resulted from the practical challenges of examining young children with primary congenital glaucoma, including limited cooperation, media opacity, and inability to obtain technically reliable OCT or UBM images.
